# Toward sustainable desalination using food waste: capacitive desalination with bread-derived electrodes†

**DOI:** 10.1039/d0ra10763h

**Published:** 2021-03-05

**Authors:** Adam R. Wood, Raghav Garg, Tzahi Cohen-Karni, Alan J. Russell, Philip LeDuc

**Affiliations:** Department of Mechanical Engineering, Carnegie Mellon University Pittsburgh Pennsylvania 15213 USA prl@andrew.cmu.edu +1 412-268-2504; Department of Engineering, Saint Vincent College, Latrobe Pennsylvania 15650 USA; Department of Material Science and Engineering, Carnegie Mellon University Pittsburgh Pennsylvania 15213 USA; Department of Biomedical Engineering, Carnegie Mellon University 5000 Forbes Avenue Pittsburgh Pennsylvania 15213 USA alanrussell@cmu.edu +1412-268-9607; Departments of Chemical Engineering, Carnegie Mellon University Pittsburgh Pennsylvania 15213 USA; Departments of Chemistry Engineering, Carnegie Mellon University Pittsburgh Pennsylvania 15213 USA; Department of Biological Sciences, Carnegie Mellon University Pittsburgh Pennsylvania 15213 USA; Department of Computational Biology, Carnegie Mellon University Pittsburgh Pennsylvania 15213 USA

## Abstract

Each year approximately 1.3 billion tons of food is either wasted or lost. One of the most wasted foods in the world is bread. The ability to reuse wasted food in another area of need, such as water scarcity, would provide a tremendous sustainable outcome. To address water scarcity, many areas of the world are now implementing desalination. One desalination technology that could benefit from food waste reuse is capacitive deionization (CDI). CDI has emerged as a powerful desalination technology that essentially only requires a pair of electrodes and a low-voltage power supply. Developing freestanding carbon electrodes from food waste could lower the overall cost of CDI systems and the environmental and economic impact from food waste. We created freestanding CDI electrodes from bread. The electrodes possessed a hierarchical pore structure that enabled both high salt adsorption capacity and one of the highest reported values for hydraulic permeability to date in a flow-through CDI system. We also developed a sustainable technique for electrode fabrication that does not require the use of common laboratory equipment and could be deployed in decentralized locations and developing countries with low-financial resources.

## Abstract 2

Sustainable approach to fabrication of capacitive desalination electrodes using food waste (bread).

## Graphical abstract

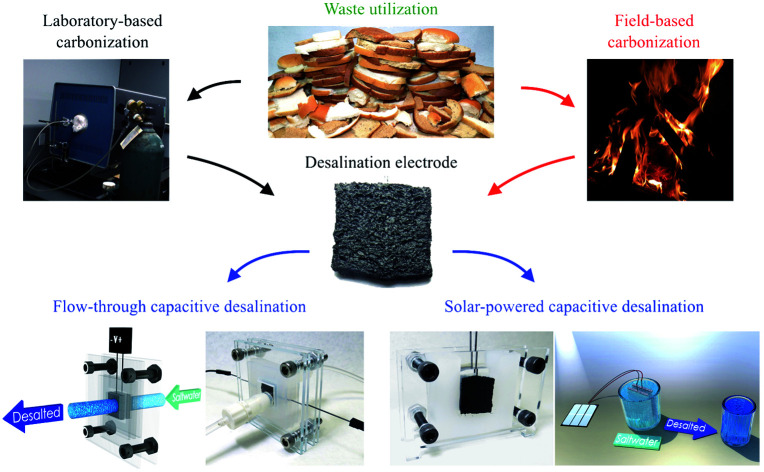

## Introduction

Meeting the water demands of a growing global population is one of the world's “grand challenges”.^1,2^ To increase freshwater supply, many areas of the world generate drinking water through desalination of brackish water and seawater.^3^ In resource-rich regions, technologies such as reverse osmosis and thermal distillation are feasible solutions for desalination. However, the development of more cost-effective and energy-efficient technologies remains an important area of research, which has led to the emergence of capacitive deionization (CDI).^4–6^

CDI desalinates a salty feed solution by electrostatically adsorbing ionic species to a pair of oppositely charged electrodes. For static CDI systems, the electrodes can be configured such that the feed stream either flows by (FB) the electrodes or flows through (FT) the electrodes.^5,6^ In FB systems, the feed stream is directed through a separator layer between the electrodes that is typically 200–500 μm thick.^7^ FT systems offer an advantage of minimizing the separator layer thickness (*i.e.* down to ∼10 μm) because it is not the primary channel for fluid transport. The reduction in separator layer thickness can enable higher desalination rates, greater reductions in feed concentration, and greater accessibility to the entire electrode surface for ion adsorption.^7–9^ Unfortunately, one of the major drawbacks of FT systems is that they typically require greater feed pressures to sustain a throughput comparable to FB systems.^7,10^ To address this issue, work has been directed towards the fabrication of hierarchical-structured electrodes possessing both large pores (0.1–200 μm diameter) to facilitate fluid transport and small pores (∼1 nm) to enhance surface area and ion adsorption capacity.^10–13^ Although past studies have shown great success, further work to investigate an easy, low-cost method towards fabricating such specialized electrodes was needed.

A major aspect of CDI research has focused on the fabrication of high surface area carbon-based electrodes.^14,15^ Carbon aerogels,^16,17^ carbon nanotubes,^18,19^ graphene-based materials,^20,21^ and activated carbons^22,23^ (ACs) are all exciting developments with the goal to combine high surface area with robust performance. ACs derived from biomass or waste products are an attractive and commonly used core component of CDI systems.^5^ However, common electrode fabrication techniques using ACs require additional materials such as binders and current collecting mesh which can increase overall electrical resistance and material cost.^24–26^ Reducing the material cost of the electrodes is a primary concern in CDI systems because it can be the largest expense in long-term full-scale operation.^26^ Several recent studies have investigated the use of binder-free, freestanding electrodes to circumvent these issues.^25,27,28^ We recently showed that an intact natural material, mangrove roots, could be carbonized and used as a FT-CDI electrode.^29^ Although our results were exciting, the global supply of mangroves and their importance in natural habitats limits their applicability. We have therefore been searching for an ideal source material that is widely available and comprised of a porous architecture so that it could be used as a freestanding FT-CDI electrode.

Globally, over 1 billion tons of food is wasted or lost on an annual basis.^30^ 44% of global food waste and loss comes from developing countries.^31^ Cereals are the most wasted foods in the world by caloric content^32^ and bread waste is one of the largest contributors to the environmental impact of supermarkets.^33^ In some regions, the amount of bread wasted in retail is comparable to waste occurring in households,^34^ which presents an opportunity for obtaining large quantities of the material in an intact condition. We hypothesized that bread would be an ideal material from which to generate a CDI electrode based on its high content of carbon due to plant-based ingredients, inherently porous microstructure, and tremendously high availability. Previous studies have generated carbon electrodes from bread, but none for CDI. Yuan *et al.* generated a lightweight carbon foam from bread and demonstrated its use for electromagnetic interference shielding.^35^ Bread has also been used to develop electrodes including for supercapacitors,^36,37^ microbial fuel cells,^38^ carbon foam for adsorption of methylene blue,^39^ and a solar absorber for steam generation.^40^

**Fig. 1:**
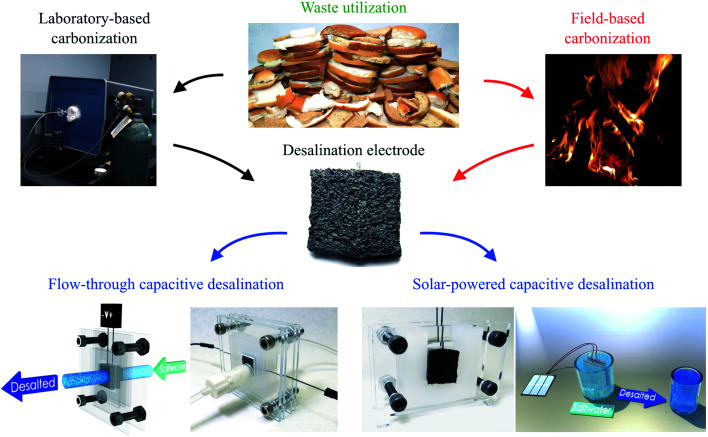
Utilization of bread for water desalination. Conductive freestanding carbon electrodes were fabricated from bread using a common laboratory-based technique and a non-laboratory-based fire-based approach. Thermal treatment with both techniques yielded high surface area carbon electrodes which were then used for desalination in a capacitive deionization (CDI) system. The hierarchical pore structure of the freestanding bread electrodes enabled high salt adsorption and low resistance to water permeation in a flow-through (FT)-CDI system. Also, coupling the bread electrodes fabricated by fire with solar energy suggests a feasible solution to water desalination in economically burdened and remote regions.

Herein, we report the development of a bread-based desalination system. We fabricated freestanding bread electrodes with a hierarchical pore structure using both a conventional laboratory technique and a decentralized point-of-use technique with controlled heating from firewood (Fig. 1). The bread-derived electrodes exhibited a competitive desalination performance in a FT-CDI system and one of the highest values for hydraulic permeability reported to date. We also demonstrated the potential for desalination in decentralized locations through coupling our firewood-based bread electrodes to inexpensive solar cells.

## Results and discussion

### Synthesis of freestanding bread-derived electrodes with high surface area using conventional laboratory techniques

**Fig. 2:**
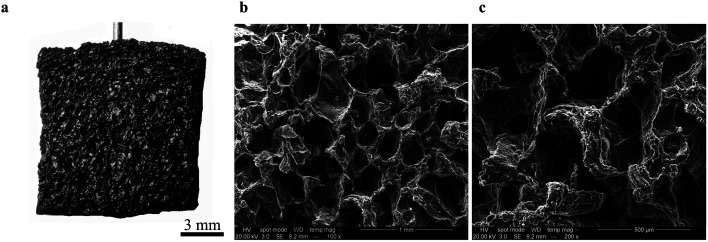
Images of freestanding bread-derived electrodes. (a) Digital image of a carbonized bread electrode. (b and c) Scanning electron microscope (SEM) images of carbonized freestanding bread electrodes showing their highly porous structure.

Our first objective was to create freestanding carbon electrodes derived from bread using conventional laboratory-based methods. We carbonized bread at 800 °C for 1 hour in a tube furnace at a ramp rate of 5 °C min^−1^ under the flow of an inert gas supply. A digital image of the carbonized material is shown in Fig. 2a. The carbonized bread was intact/freestanding and had a structural appearance that was very similar to the bread before carbonization (Fig. S1†); the findings are supported by others who have utilized bread.^35,36^ Scanning electron microscopy (SEM) revealed that the carbonized bread was comprised of a large interconnected pore network (Fig. 2b and c) which could potentially enable low-resistance to water transport in a FT system.

**Fig. 3:**
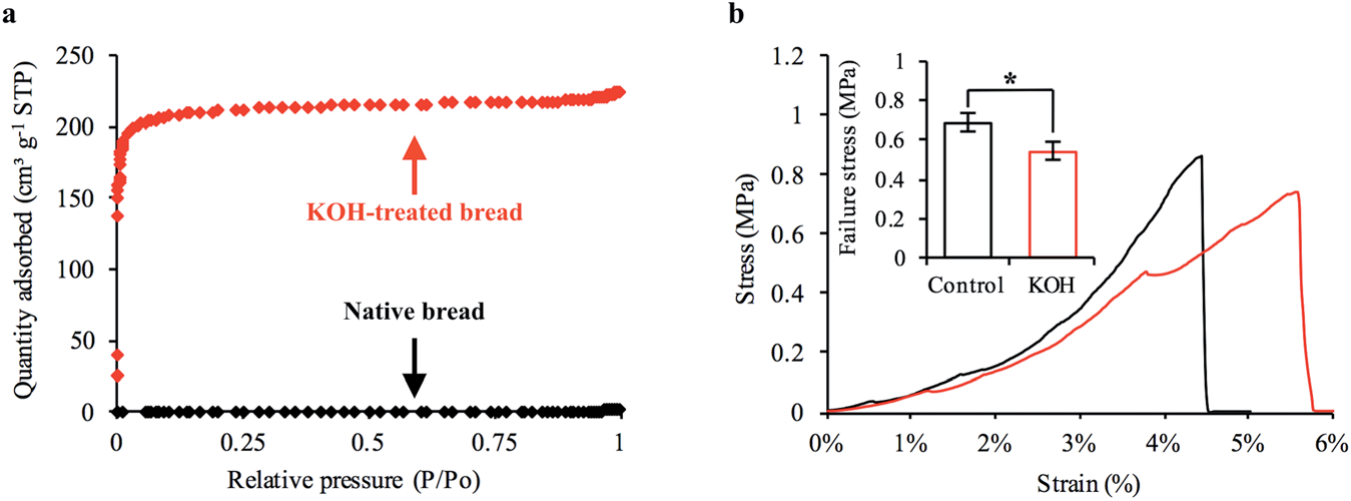
Characterization of bread-derived electrode surface properties and structural stability. (a) Nitrogen adsorption isotherm for intact bread electrodes before (black diamonds) and after KOH treatment (red diamonds). (b) Representative stress-strain curve obtained from low-level compressive testing with freestanding bread electrodes before (black line) and after KOH treatment (red line). Inset is the failure stress for non-activated and KOH-activated bread electrodes (mean ± s.e.m.; *p < 0.05).

The specific surface area of CDI electrodes is one of the primary factors that influences desalination performance.^5^ It is typically desirable to have high surface area materials as this usually leads to a high capacity for salt adsorption.^14^ To characterize the surface properties of the carbonized bread we used nitrogen adsorption and Brunauer–Emmet–Teller (BET) analysis. BET analysis of the gas adsorption isotherm (Fig. 3a) revealed that the specific surface area of the native carbonized bread was approximately 1 m^2^ g^−1^, which was 2–3 orders of magnitude less than that of high-performance CDI electrodes.^15^ To increase the specific surface area of the carbonized bread, we used potassium hydroxide (KOH) activation. Briefly, chemical reactions between KOH and carbon at elevated temperature resulted in surface etching.^41^ The surface etching introduced porosity on the order of nanometers and ultimately led to an increased specific surface area of the material. Carbonized bread was soaked in a 3.5 M KOH solution for 2 hours and then immediately heated to 800 °C for 1 hour at a ramp rate of 5 °C min^−1^ in an inert atmosphere. After thoroughly washing the KOH-treated sample, the surface properties of the carbonized bread were once again investigated using nitrogen adsorption and BET analysis. In Fig. 3a, the increased quantity of adsorbed gas at similar partial pressures for the KOH-treated bread in comparison to the native bread indicated a higher specific surface area. BET analysis determined the specific surface area of the freestanding KOH-treated bread to be over 650 m^2^ g^−1^. The total pore volume of the native bread and KOH-treated bread were approximately 0.004 cm^3^ g^−1^ and 0.346 cm^3^ g^−1^, respectively. The DFT micropore volume for the native bread electrodes was not detectable and the mesopore volume was 0.003 cm^3^ g^−1^. The DFT micropore and mesopore volume for KOH-treated bread electrodes was 0.306 cm^3^ g^−1^ and 0.005 cm^3^ g^−1^, respectively.

### Synthesis of freestanding bread-derived electrodes with high surface area using field-based techniques

High-cost laboratory equipment used in electrode fabrication can, unfortunately, limit applicability. This lead us to develop an alternative fabrication process that could be realized in decentralized locations and coupled with solar energy to achieve desalination. Drawing inspiration from the thousands of years old process of making charcoal, we hypothesized that we could use a firewood-based approach to fabricate freestanding bread-derived electrodes. One previous report had investigated a similar method to making charcoal for the fabrication of bread-based solar absorbers.^40^ However, thermal treatment was only conducted at approximately 400 °C and no electrochemical studies were conducted. Furthermore, pyrolysis at 400 °C typically results in materials with ultrahigh electrical resistivity,^42^ which is not conducive to CDI. In our initial efforts, we positioned bread samples in the middle of a relatively high-temperature fire to achieve carbonization (Fig. S2a†). Although we had approximate temperature estimates of our wood-based fire using an IR thermometer, we were not able to obtain the exact temperature measurements because the IR thermometer's maximum temperature measurement was 750 °C; previous work has shown wood-based fire temperatures exceed 800 °C.^43^ Ideally, oxygen surrounding the bread samples would be consumed in the combustion process of wood, which would create an oxygen depletion zone around the bread samples and induce thermal decomposition of the material instead of burning. We were able to successfully produce bread-derived electrodes with low electrical resistance (*R* ∼ 10 ohms) using this firewood-based technique. However, we were not able to retain the freestanding structure of bread to the degree we had previously achieved in the laboratory as the samples experienced warping and cracking (Fig. S2b†).

The heating rate when carbonizing natural materials can affect the structural integrity of the final product; for example, if the heating rate is too fast, this can create a non-uniform temperature profile throughout the material, which can then generate internal stresses and induce cracking.^44^ We estimated that the heating rate used in our fire-based approach was at least 100 times faster than what was used in the lab, and therefore hypothesized that this was the cause of warpage/cracking. To reduce the heating rate, we controlled the intensity of the fire and the sample proximity to the fire to create a gradual, relatively low-temperature treatment (Fig. S3a†). After approximately 1 hour, the bread samples remained intact and exhibited minimal signs of warpage and cracking (Fig. S3b†). We had to solve a challenge as the samples exhibited extremely high electrical resistance (*R* > 10^7^ ohms). Previous work has shown that the electrical resistance of natural-based materials can decrease by more than 10 orders of magnitude between the carbonization temperatures of 600 °C and 800 °C.^42^ We therefore continued to increase the intensity of the fire to that of our original high-temperature treatment (Fig. S3c†). After 1 hour of relatively high-temperature treatment, the samples exhibited minimal warpage/cracking (Fig. S3d†) and had an electrical resistance on the same order of magnitude as the samples fabricated in the controlled laboratory furnace (*R* ∼ 10 ohms).

Similar to our previous results in the lab, the native fire-based electrodes exhibited a very low specific surface area (less than 1 m^2^ g^−1^). To chemically activate the electrodes, we soaked them in KOH, which can be obtained from wood ashes in a direct approach,^45^ for 2 hours and exposed the samples to another relatively high temperature fire treatment for 1 hour. Following treatment, the electrodes exhibited a specific surface area as high as 800 m^2^ g^−1^ (Fig. S4†). We used Raman spectroscopy to investigate if the molecular structure of the firewood-based electrodes differed from the laboratory-based electrodes. Both electrodes exhibited peaks in the Raman spectra at ∼1352 cm^−1^ and ∼1598 cm^−1^ which correspond to the defect (D) and graphitic (G) bands of carbon, respectively (Fig. S5†). Interestingly, the ratio of disordered-to-graphitic carbon (*I*_D_/*I*_G_) in the electrodes differed only slightly between carbonization methods. The *I*_D_/*I*_G_ values for the firewood-based and laboratory-based electrodes were 1.07 and 1.08, respectively, and very similar in magnitude to other studies using naturally-derived materials.^46^ The yield from laboratory-based and field-based fabrication were approximately 5 g and 40 g of electrode material, respectively. The low yield produced from laboratory-based fabrication was due to size restrictions of the tube furnace. Importantly, we hypothesize that the yield from field-based fabrication could have easily been increased, but was not specifically investigated in this study.

### Evaluating structural stability of freestanding bread-derived electrodes

The porosity and density of bread has been reported to influence the resulting mechanical properties of the carbonized structure.^35^ Bread electrodes with relatively large pores and low density exhibit lower mechanical strength in comparison to higher density electrodes with small pores.^35^ The KOH-treated bread developed with laboratory techniques had a structural appearance (Fig. S6a–c†) that was almost identical to that of native bread (Fig. 2a–c) and a similar surface appearance on the order of microns (Fig. S7a and b†). However, one side effect of KOH treatment is that material loss through etching is inevitable. For example, the total dry-mass of bread retained through initial carbonization was 26%. After chemical activation, the total dry-mass of bread retained dropped to 20%. We were concerned that material loss would have adverse effects on the structural stability of the freestanding electrodes. If severe, the bread-derived electrodes were too brittle to handle and introduce into a FT-CDI system.

To investigate this issue, we mechanically tested native and KOH-treated bread that was developed with laboratory techniques. Our goal was to determine the average compressive stress at which each sample experienced brittle fracture. A representative stress–strain curve for the native and KOH-treated bread is shown in Fig. 3b. The sharp drop-off in each representative curve was the compressive stress at which brittle fracture occurred. We found that KOH treatment reduced the mechanical strength of the freestanding bread electrodes. The native and KOH-treated samples had an average failure stress of 0.69 MPa and 0.55 MPa, respectively (Fig. 3b inset). Although chemical activation reduced the mechanical strength of the freestanding electrodes, the magnitude at which failure occurred indicated that the KOH-treated bread was sufficiently strong. A KOH-treated freestanding electrode with a 6 × 6 mm square cross-section could theoretically support a mass of 2 kg.

### Performance characterization of bread-derived electrodes in a FT-CDI system

We hypothesized that our freestanding bread electrodes would be ideal for a FT-CDI system. The large interconnected pore structure on the order of 10 s to 100 s of microns could facilitate high fluid transport while the high specific surface area could enable high salt adsorption. We characterized the water transport properties of the freestanding carbonized bread by constructing a FT cell to house the electrodes while measuring the pressure differential across the cell at varying flow rates (Fig. S8 and S9†). We used Darcy's law to calculate the average hydraulic permeability of the carbonized bread to be 2.32 × 10^−11^ m^2^, which is one of the highest reported values for FT-CDI electrodes^10,29^ and 5 orders of magnitude greater than the reported value for a commercially available porous carbon electrode.^10^

**Fig. 4:**
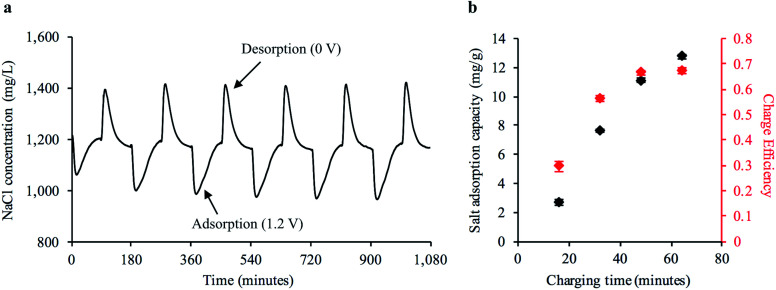
Desalination performance of freestanding bread electrodes in a FT-CDI system. (a) Adsorption–desorption curve for single pair of bread electrodes operated cyclically in FT-CDI system. The sharp decrease in NaCl concentration relative to the stock solution of 1200 mg L^−1^ was ions being adsorbed to the electrodes from the feed stream when a potential of 1.2 V was applied. The increase in NaCl concentration relative to the stock concentration was ions being released back into solution when the voltage potential was removed. (b) Average salt adsorption capacity (SAC) and cumulative charge efficiency during the adsorption stage of cyclic CDI operation for a single pair of bread electrodes (mean ± s.e.m., *n* = 6 cycles).

Next, we investigated the cyclic desalination performance of the bread electrodes in a FT-CDI system over the course of 18 hours using the cell design shown in ESI Fig. S10.† A stock NaCl solution of 1200 mg L^−1^ was continuously pumped through the FT-CDI cell while charge–discharge potentials of 1.2–0 V were applied to the bread electrodes. The salt concentration was monitored downstream of the FT-CDI cell using a flow-through conductivity meter. Fig. 4a shows the change in salt concentration during cyclic CDI operation. The sharp decrease in salt concentration relative to the stock solution was due to ion adsorption to the carbonized bread when 1.2 V was applied to the electrode pair. As the electrode surface became saturated with ions, the concentration gradually increased back to the concentration of the stock solution. The adsorbed ions were then released back into the flowing stock solution when the potential was removed, which generated the brine stream portions shown in Fig. 4a (*i.e.* higher salt concentration relative to stock). The corresponding current response and voltage profile for Fig. 4a is shown in ESI Fig. S11.†

To compare the performance of the freestanding bread-derived electrodes with other CDI electrodes we examined a range of factors including salt adsorption capacity (SAC) and charge efficiency (*Λ*) during cyclic operation. The SAC is defined as the ratio of salt adsorbed from the feed stream to the mass of the electrode pair.^47^ Values for SAC typically range from 2 mg g^−1^ to as high as 21 mg g^−1^ for FT electrodes and naturally-based electrodes,^29^ although other studies have also reported lower values.^14^*Λ* is the ratio of salt adsorption to charge transferred and is indicative of energy consumption.^47^ Values for *Λ* typically range from 0.5–0.8 for CDI systems.^48^ Significantly lower *Λ* values compared to the theoretical maximum (unity) for CDI can be caused by co-ion expulsion during the charging process.^12^ Values closer to the theoretical maximum (unity) have been achieved with the use of ion specific membranes that limit co-ion expulsion,^49^ but also result in higher material cost.^26^Fig. 4b shows the average SAC at representative time points during the adsorption phase from at least six continuous charge–discharge cycles. We observed an average SAC as high as 12.7 mg g^−1^ at 1.2 V with a corresponding *Λ* of 0.67, which is competitive with recently reported values for FT electrodes, naturally-based electrodes, and freestanding CDI electrodes. Recent reports for freestanding CDI electrodes developed from basswood^25^ and commercially available AC^27^ have observed a SAC of 5.7 mg g^−1^ and 8.9 mg g^−1^ with a *Λ* of 0.30 and 0.48, respectively.

We also calculated the areal and volumetric SAC for our bread-derived electrodes. The areal SAC is the ratio of adsorbed salt to the available surface area of the electrodes.^25,27^ For clarification, this is not the specific surface area of the electrodes, but rather the general surface area of the electrode surface that contributes to salt adsorption. The volumetric SAC is the ratio of adsorbed salt to the volume of the electrode pair.^47^ The observed areal and volumetric SAC for the bread electrodes was 0.23 mg cm^−2^ and 2.5 mg cm^−3^, respectively. The areal and volumetric SAC of our bread electrodes is also competitive with recently reported values for freestanding CDI electrodes.^25,27^ For calculating areal SAC, we used the inner and outer surfaces of the electrode pair, along with the side surfaces. Although other studies typically only consider the inner electrode surfaces, we cannot assume the outer and side surfaces do not contribute to salt adsorption during FT operation. One of the primary advantages of the bread-derived electrodes is that they did not require the use of common fabrication materials (*i.e.* binders, conductive additives, current collecting mesh, *etc.*). Hand *et al.* estimated that the electrodes were the single greatest cost factor in long-term operation of full-scale CDI systems. Therefore, the development of low-cost electrodes from bread could be a promising avenue to reduce capital cost of CDI systems while lowering the economic footprint of food waste.

**Fig. 5:**
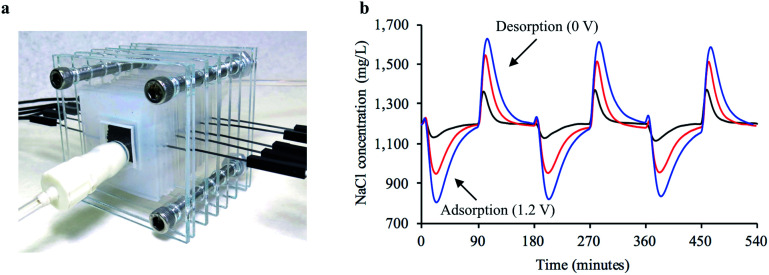
Desalination performance of serially arranged freestanding bread electrodes in a FT-CDI system. (a) Representative image of FT-CDI system housing 5 pairs of bread electrodes in series. (b) Adsorption–desorption curves for charging one (black line), three (red line), and five (blue line) pairs of bread electrodes arranged in series with an applied potential of 1.2 V.

We also investigated a method to increase the reduction in feed concentration while only processing the feed solution once, which is closely related to CDI applications.^5^ We hypothesized that closely arranging a number of pairs of electrodes in series would produce an additive desalination effect that would lead to higher reductions in feed concentration. Freestanding bread electrodes are particularly advantageous for a serial arrangement because of their high hydraulic permeability, whereas electrodes with low hydraulic permeability could drastically raise required feed pressures. We designed a compact FT-CDI cell which housed five sets of electrode pairs arranged in series (Fig. 5a). A charge–discharge potential of 1.2–0 V was applied to either 1, 3, or 5 sets of electrode pairs while a 1200 mg L^−1^ NaCl solution was continuously passed through the cell. Simultaneously charging more electrode pairs in series led to larger reductions in feed concentration (Fig. 5b).

### Solar-powered desalination with firewood-based bread electrodes

**Fig. 6:**
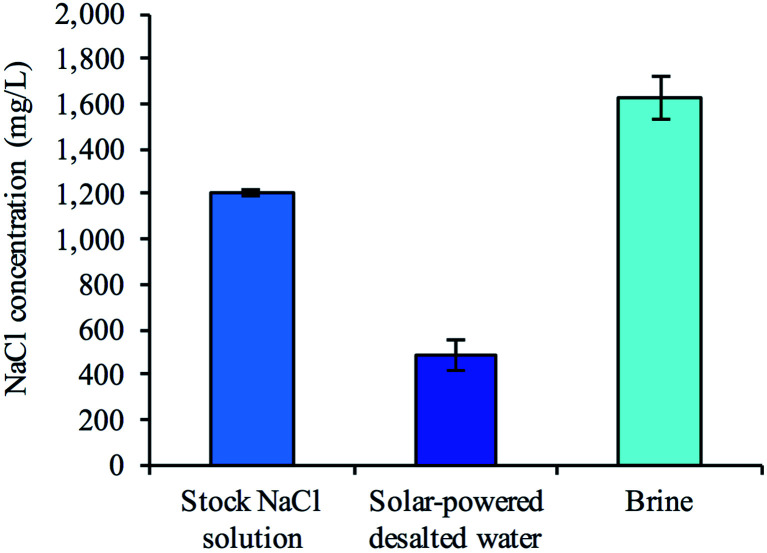
Desalination performance of decentralized bread-derived CDI system fabricated using firewood-based approach. The freestanding bread electrodes were placed in an unpalatable salty solution (1200 mg L^−1^ NaCl) and attached to a solar cell (rated at 1.5 V). After 2.5 hours of solar-powered CDI, approximately 56% of the total volume was removed. The NaCl concentration was reduced from 1200 mg L^−1^ to 490 mg L^−1^. An equivalent volume of stock solution was added to the CDI cell and no potential was applied for 2 hours. Ion desorption from the electrode surfaces generated a brine solution, which indicated the potential to reuse the electrodes (mean ± s.e.m.; *n* = 4).

To examine whether a decentralized desalination system could come from bread and a simple solar cell, we tested the desalination capabilities of the firewood-based bread-derived electrodes in a CDI system powered by solar energy. A pair of bread electrodes were placed in a reservoir of unpalatable salty water (1200 mg L^−1^) and connected to a 1.5 V solar cell exposed to outside light. Solar CDI experiments were conducted in Pittsburgh, PA, USA on 3 different days during February of 2019. After charging the solar-powered CDI system for 2.5 hours, over half of the total reservoir volume (56% on average) was recovered and tested to determine water salinity. The average recovered salt concentration was 490 mg L^−1^ (Fig. 6). The calculated sodium concentration was reduced from 472 mg L^−1^ to approximately 193 mg L^−1^, which is below the guidelines set by the World Health Organization for esthetic considerations.^50^ This does not though reach a lower value for specific individuals who are on a restricted sodium intake, which is 20 mg per L per day. Overall, based on recommendations for daily water intake^51^ (2.7 L for women and 3.7 L for men) and maximum daily sodium intake^52^ (2300 mg per day), the sodium ingested from our desalinated solution would only contribute 23% to the recommended value for women and 31% for men. Importantly, a brine solution was generated in each trial after disconnecting the electrode pair from the solar cell. This indicated that the adsorbed salt ions were being released from the electrode surface and that the electrodes could be reused for desalination.

## Conclusions

We created an approach to desalinate water using naturally-derived waste material (bread) through controlled carbonization. The carbonized structures had an advantageous pore structure and produced highly permeable, freestanding FT-CDI electrodes. We observed an average salt adsorption capacity of 12.7 mg g^−1^ during cyclic operation at 1.2 V and a corresponding charge efficiency of 0.67. The measured hydraulic permeability of the bread-derived electrodes (2.32 × 10^−11^ m^2^) was one of the highest reported values to date and presented the opportunity for multiple electrode pairs to be arranged in series without dramatically raising the resistance to water flow. Compact serial arrangement of multiple electrode pairs in a FT-CDI system enabled larger reductions in salt concentration during continuous flow operation.

We also used these scientific findings to create a new technique for electrode fabrication that does not require the use of common laboratory equipment and can be globally deployable even to decentralized locations. Using a firewood-based approach, we fabricated CDI electrodes from bread and specifically demonstrated a gradual heating technique to maintain the structural integrity of the freestanding electrodes. We then coupled the firewood-based electrodes to a low-cost solar cell and reduced the salt concentration of 1200 mg L^−1^ brackish water by nearly 60% with a recovery yield of 56%. These findings will have impacts ranging from the development of low-cost hierarchical-structured electrodes using naturally-derived renewable materials to desalination in developing and decentralized regions of the world.

## Methods

### Electrode fabrication (laboratory-based)

Bread (Pepperidge Farm® Stone Ground 100% whole wheat) was cut into 20 × 20 × 4 mm cubes and 60 mm long graphite rods (0.5 mm diameter) were inserted into the samples. The bread blocks were then dried at 80 °C between two pieces of acrylic for approximately 4 hours. The samples were carbonized in a Lindberg 54553-VH tube furnace at 800 °C for 1 hour under 95–100 cm^3^ min^−1^ nitrogen flow at a heating rate of 5 °C min^−1^. Following carbonization, the samples were washed with DI water 3 times and dried at 80 °C for at least 3 hours. The samples were then soaked in a 3.5 M KOH solution for 2 hours at a KOH : sample mass ratio of 3.5 : 1. Immediately following KOH treatment the samples were heated to 800 °C for 1 hour under 95–100 cm^3^ min^−1^ nitrogen flow at a heating rate of 5 °C min^−1^. The samples were thoroughly rinsed by flowing DI water through the porous carbon structure. The final dimensions of the electrodes were approximately 12.7 × 12.7 × 2.6 mm.

### Electrode fabrication (fire-based)

The bread was cut into 8 mm thick blocks with a (width) × (length) ranging from 20 × 20 mm to 75 × 62.5 mm. Then, 100 mm long graphite rods (0.7 mm diameter) were inserted into the bread blocks. The samples were put into a metal container with a small hole (∼1 cm diameter) in the top of it and placed in the middle of a square-lined fire for 1 hour (Fig. S3a†). Firewood was added around the samples to increase temperature and minimize oxygen content (Fig. S3c†). The temperature of the fire was measured with an IR thermometer and determined to be at least 750 °C. However, the measured temperature was at the upper limit of the IR thermometer range. Previous work has shown that the temperature of wood-based fires can exceed 800 °C.^43^ After 1 hour, the samples were recovered from the fire and the metal can that housed the samples was immediately placed top down on the ground. This was done to minimize the amount of oxygen diffusing into the can through the small hole in the lid. The samples were washed with DI water 3 times and dried at 80 °C for at least 3 hours. The samples were then soaked in a 3.5 M KOH solution for 2 hours at a KOH : sample mass ratio of 3.5 : 1. Immediately following KOH treatment the samples were again heated in the high temperature fire for 1 hour (Fig. S3c†). Following thermal treatment, the samples were thoroughly rinsed by flowing DI water through the porous carbon structure.

### Characterization

The structure of the freestanding carbonized bread was characterized by scanning electron imaging (SEM) on an FEI Quanta 600 FEG SEM. Specific surface area was determined by the Brunauer–Emmet–Teller (BET) method for nitrogen adsorption isotherm using a Micromeritics Gemini VII Surface and Porosity instrument (*n* = 3 for each condition). Pore size distribution was determined using nonlocal density functional theory (NLDFT). Raman spectroscopy was performed by NT-MDT NTEGRA spectra. The acquisition time was 30 seconds, the laser power was 2.38 mW, and the excitation was 532 nm (*n* = 3 for each condition). Mechanical analysis was examined using a TA Instruments RSA-G2 Solids Analyzer with an axial loading rate of 20 μm s^−1^. Compressive failure strength was classified by a sharp drop off in the stress–strain curve (Fig. 3b) (*n* = 9 for each condition).

The applied pressure needed to retain a defined flow rate through the bread electrodes was examined using the setup shown in ESI Fig. 8a.† The flow rate was controlled through using a syringe pump (New Era Pump System Inc., Model NE-300) and the upstream pressure was measured through a pressure sensor (Honeywell ASDX005G24R) mounted to an Arduino UNO R3. The output voltage from the pressure sensor was correlated to applied pressure through a calibration curve obtained immediately before testing (Fig. S8b†). Three sets of six carbonized bread electrodes arranged in series were used for testing. The permeation area was approximately 100 mm^2^ and the average thickness of each electrode was 2.6 mm. The hydraulic permeability was calculated according to Darcy's law:*κ* = (*QμL*)/(Δ*PA*)where *κ* is permeability (m^2^), *Q* is flow rate (m^3^ s^−1^), *μ* is dynamic viscosity (Pa s), *L* (m) is total length of the electrodes, Δ*P* (Pa) is the applied pressure differential, and *A* (m^2^) is permeation area.

### Flow-through capacitive deionization experiments

Two bread electrodes with dimensions of approximately 12.7 × 12.7 × 2.6 mm and a net mass of 165 mg were arranged in series in a FT-CDI cell (Fig. S10†). Two nylon membranes (Millipore NY20) were used as insulators to separate the electrodes by approximately 110 μm. A solution of 1200 mg L^−1^ NaCl was delivered through the electrodes at 0.3 mL min^−1^ and cycles of charging–discharging were applied at 1.2–0 V. Each cycle lasted 180 minutes with an even charge–discharge duration. The salt concentration of the solution exiting the FT-CDI cell was measured using a custom-built conductivity meter which is described in detail in Wood *et al.* 2019.^29^ The salt adsorbed was calculated by quantifying the area between the stock solution (1200 mg L^−1^) and the permeate solution during the charging phase of the CDI cycle. The SAC was calculated by dividing the salt adsorbed by the net mass of the electrodes. Charge efficiency was calculated by: *Λ* = (*ΓF*)/*Σ*, where *Γ* is the salt adsorbed (mol g^−1^), *F* is the Faraday constant (96 485 C mol^−1^), and *Σ* is charge transferred (C g^−1^).

FT-CDI experiments with multiple electrode pairs arranged in series were conducted in continuous flow mode at a rate of 0.36 mL min^−1^ with a 1200 mg L^−1^ NaCl solution. The electrode pairs had an average mass of 194 mg and were spaced approximately 1.75 mm apart in the FT-CDI cell (Fig. 5a). Nylon membranes were used as insulators between the individual electrodes in each pair and separated the electrodes by approximately 110 um. First, six charge–discharge cycles at 1.2–0 V were applied to all five electrode pairs. The total duration of each cycle was 180 minutes with an equal charge–discharge phase. The first three cycles were used to condition the electrodes. Then, the first and second electrode pairs in reference to the cell inlet were disconnected from the power supply and three charge–discharge cycles were applied to the remaining pairs. Finally, the third and fourth electrode pairs were disconnected from the power supply and three charge–discharge cycles were applied to the fifth pair.

### Solar-powered desalination with fire-based bread electrodes

Two bread electrodes with dimensions of approximately 15 × 15 × 4 mm and a net mass of 200 mg were submerged in a 3 mL reservoir of 1200 mg L^−1^ NaCl solution (ESI Fig. S12†). The electrodes were briefly degassed (60 seconds or less) to remove air and a nylon membrane was place between the electrodes for electrical insulation. The electrodes were connected to a 1.5 V solar cell (AMX3D 1.5 V; 400 mA; 80 × 60 mm) and placed outside for 2.5 hours in the afternoon during the month of February 2019 in Pittsburgh, Pennsylvania, USA. After charging, the electrodes were briefly removed from the reservoir and the remaining volume was removed from the housing device and tested to determine the NaCl concentration. An equivalent volume of stock NaCl solution (1200 mg L^−1^) was immediately inserted back into the housing device and the electrodes were placed back in the reservoir without being connected to the solar cell. After 2 hours, the salt concentration of the reservoir was characterized. The electrodes were then rinsed with DI water and used for further testing.

### Statistical analysis

All statistical analysis was conducted using a two-tailed paired Student's *t*-test. Standard error mean (s.e.m.) was calculated by dividing the sample standard deviation by the square root of the sample size.

### Data availability

The datasets generated during and/or analyzed during the current study are available from the corresponding author on reasonable request.

## Author contributions

P. L., A. J. R. and A. R. W. designed and directed the project. A. R. W. carried out most of the experiments and data analysis. R. G. and T. C. helped with some of the experiments and data analysis, and provided valuable input for experimental design. P. L., A. J. R. and A. R. W. wrote the manuscript.

## Conflicts of interest

The authors declare no competing interests.

## Supplementary Material

RA-011-D0RA10763H-s001
