# Supplementary material for: Toward sustainable desalination using food waste: capacitive desalination with bread-derived electrodes
Source: RSC Adv. 2021 Mar 5;11(16):9628–37. doi: 10.1039/d0ra10763h (PMC8695462; doi:10.1039/d0ra10763h)
Supplement: RA-011-D0RA10763H-s001 [file RA-011-D0RA10763H-s001.pdf]

Electronic Supplementary Material (ESI) for RSC Advances.
This journal is © The Royal Society of Chemistry 2021

Toward sustainable desalination using food waste: Capacitive desalination with bread-derived electrodes

Adam R. Wood[1], Raghav Garg[2], Tzahi Cohen-Karni[2,3], *Alan J. Russell[3,4,5], and *Philip LeDuc[1,3,6,7]

[1]Department of Mechanical Engineering, Carnegie Mellon University, Pittsburgh, Pennsylvania 15213.
[2]Department of Material Science and Engineering, Carnegie Mellon University, Pittsburgh, Pennsylvania 15213.
[3]Department of Biomedical Engineering, Carnegie Mellon University, Pittsburgh, Pennsylvania 15213.
[4]Departments of Chemical Engineering, Carnegie Mellon University, Pittsburgh, Pennsylvania 15213.
[5]Departments of Chemistry Engineering, Carnegie Mellon University, Pittsburgh, Pennsylvania 15213.
[6]Department of Biological Sciences, Carnegie Mellon University, Pittsburgh, Pennsylvania 15213.
[7]Department of Computational Biology, Carnegie Mellon University, Pittsburgh, Pennsylvania 15213.

*Corresponding Authors: Professor Philip LeDuc; 5000 Forbes Avenue, Pittsburgh, Pa 15213; Phone number: 412-268-2504; Email: prl@andrew.cmu.edu, Professor Alan J Russell; 5000 Forbes Avenue, Pittsburgh, Pa 15213; Phone number: 412-268-9607; Email: alanrussell@cmu.edu

Keywords: Food waste, Sustainability, Desalination

Supporting Information

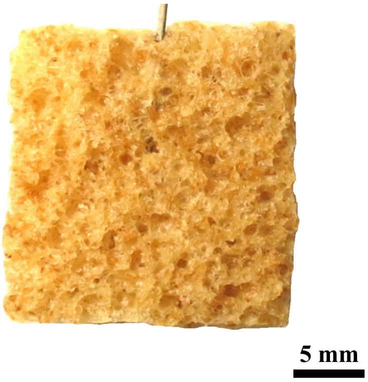

Supplementary Figure 1 - Digital image of bread before carbonization showing porous structural appearance.

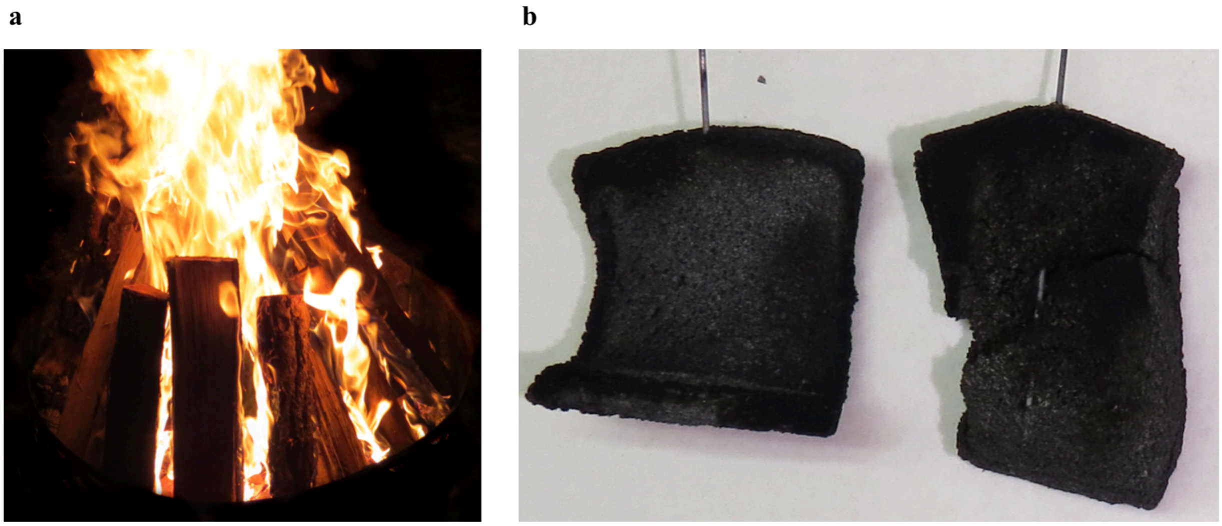

Supplementary Figure 2 - (a) Representative image of fire for thermally treating bread. The fire is arranged so that oxygen is consumed through combustion before diffusing to the center, therefore creating an oxygen depletion zone in the middle of the fire. If the bread is immediately inserted into the middle of the fire, there is no control over the heating rate, which then leads to (b) warping and cracking of the electrodes.

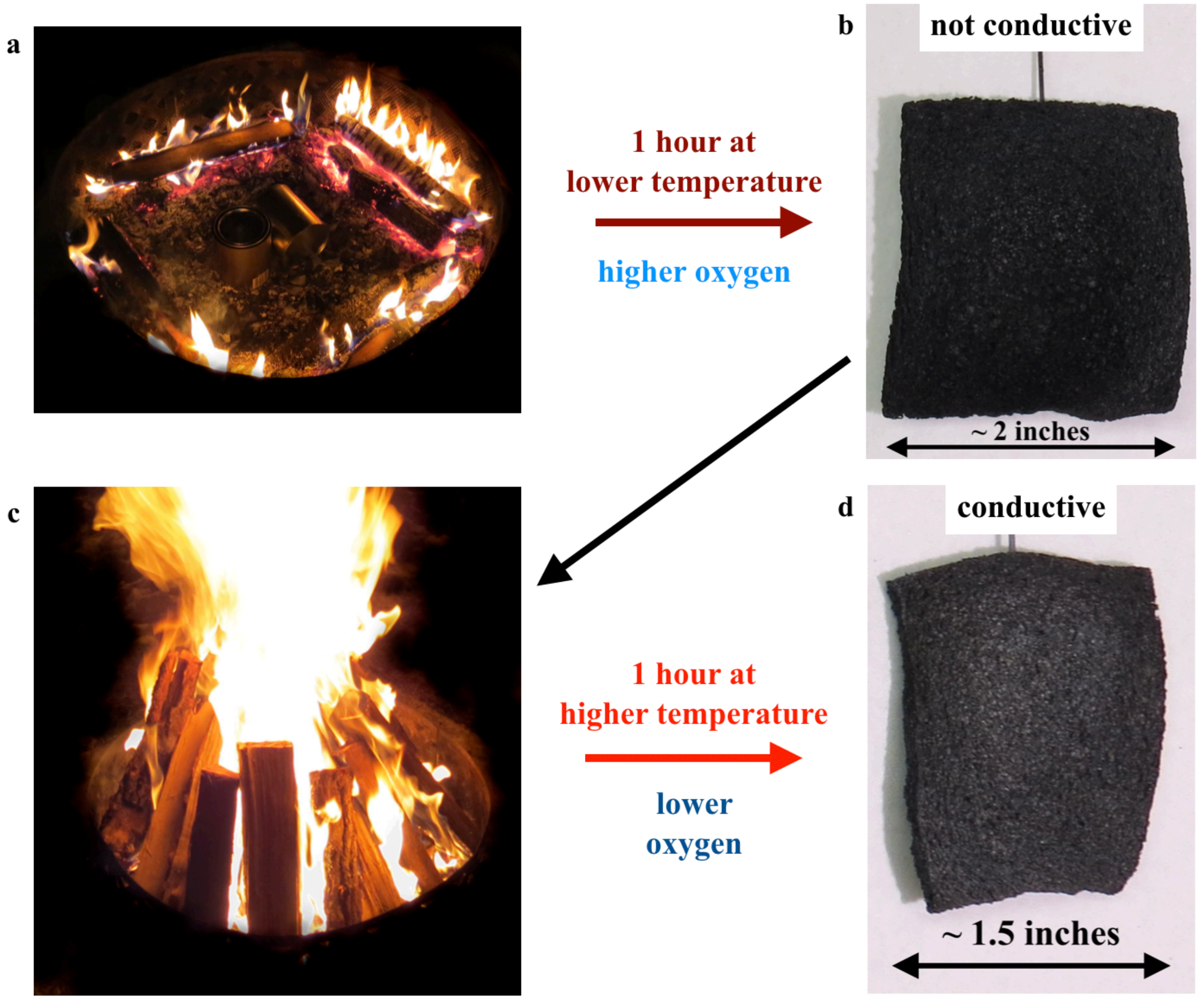

Supplementary Figure 3 - (a) Image of bread (in metal can) in the middle of a relatively low temperature fire with higher oxygen content in location of the samples. (b) Hardened bread sample after 1 hour of low temperature treatment. Sample is not electrically conductive, but lower temperature heat treatment minimizes warping and cracking. (c) A non-conductive sample is then placed in the middle of a higher temperature fire for 1 hour. Combustion consumes oxygen diffusing into the middle of the fire, creating an oxygen depletion zone around the bread samples. (d) Higher temperature treatment with lower oxygen content leads to a conductive freestanding bread electrode with minimized warping and cracking.

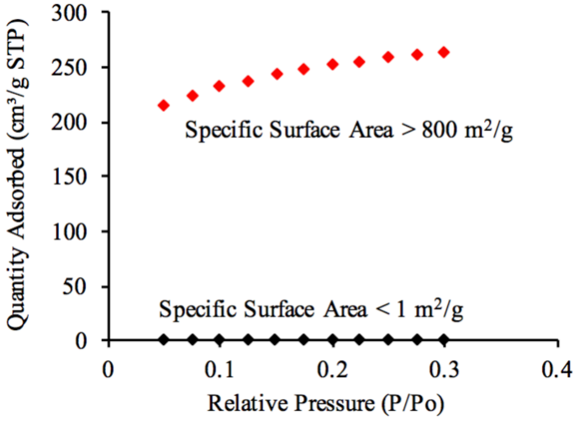

Supplementary Figure 4 - Nitrogen adsorption isotherm for intact bread electrodes before (black diamonds) and after KOH treatment (red diamonds) using fire-based thermal treatment.

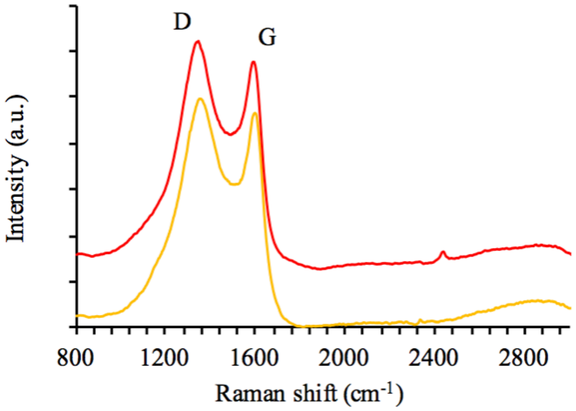

Supplementary Figure 5 - Raman spectra for KOH-activated bread electrodes fabricated using fire-based thermal treatment (orange line) and conventional laboratory equipment (red line).

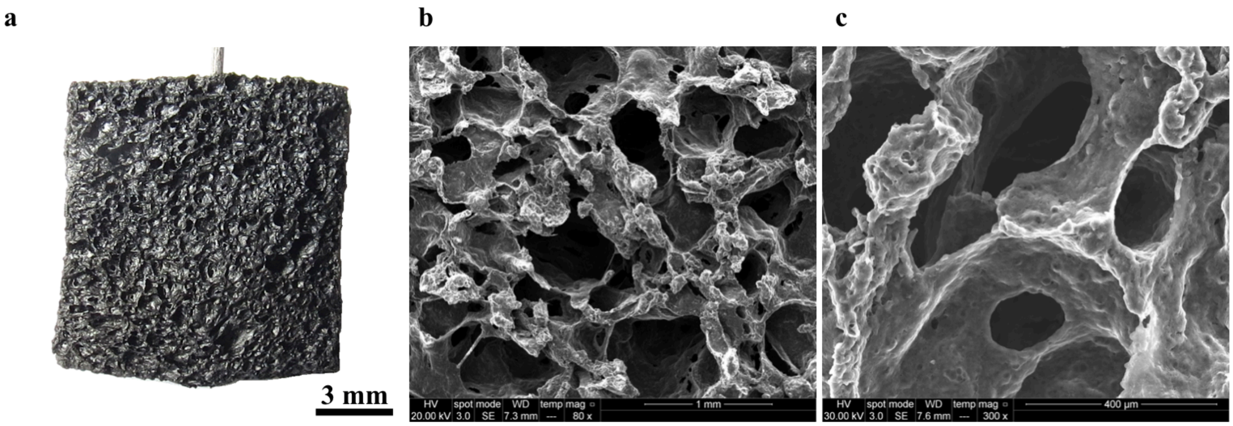

Supplementary Figure 6 - (a) Digital image of bread-derived electrode after KOH treatment. (b,c) Scanning electron microscope (SEM) images of carbonized bread electrodes after KOH treatment.

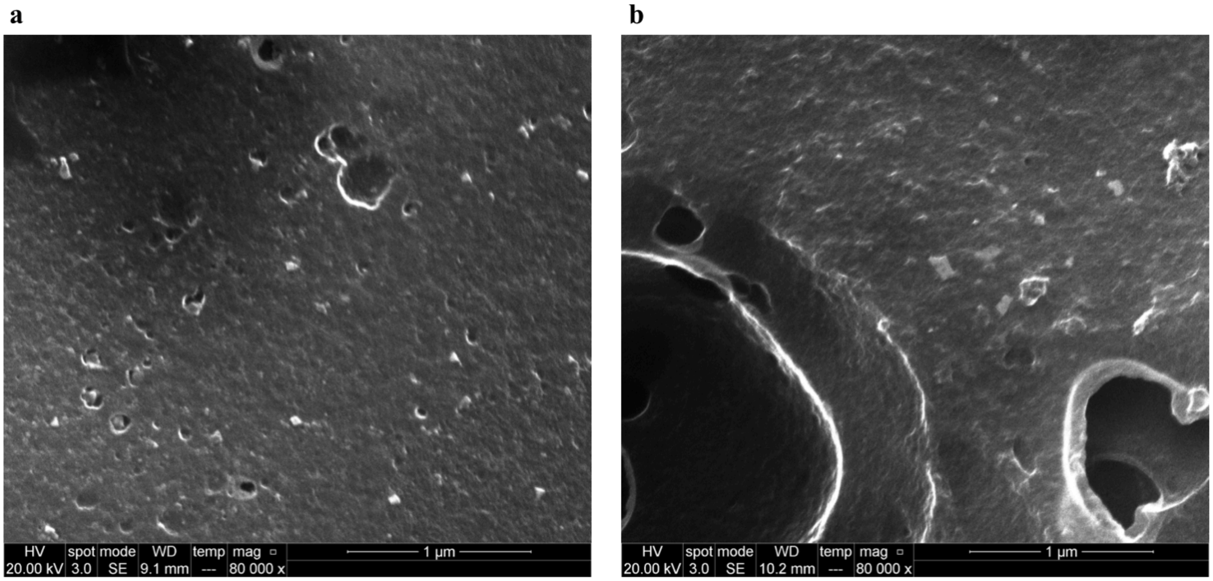

Supplementary Figure 7 - Higher magnification SEM images of carbonized bread electrodes (a) before KOH treatment and (b) after KOH treatment.

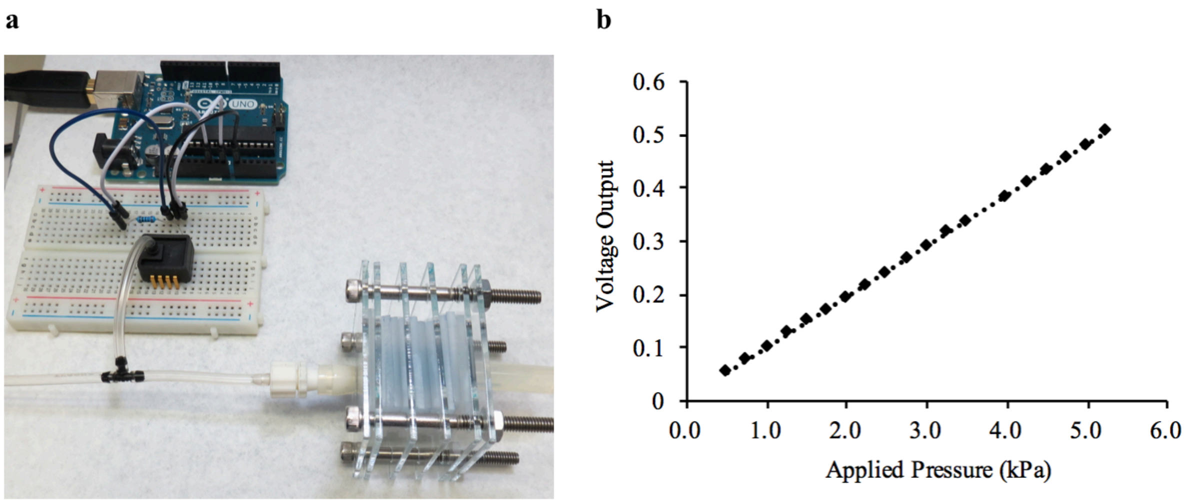

Supplementary Figure 8 - (a) Experimental setup for determining the pressure differential across the FT-CDI cell at varying flow rates. The voltage output at varying flow rates was correlated to an applied pressure by the (b) calibration curve obtained before testing.

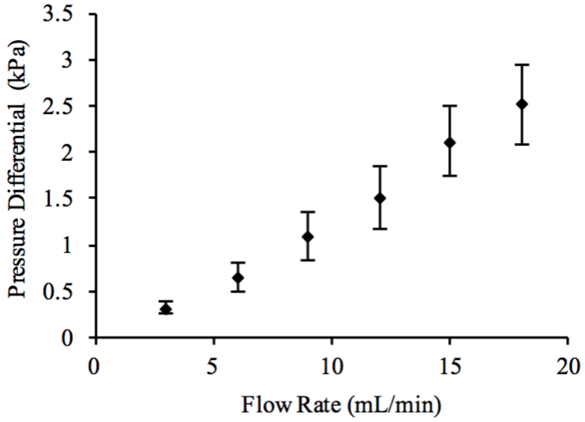

Supplementary Figure 9 - Pressure differential across FT-CDI cell with three pairs of bread electrodes arranged in series for varying flow rates (mean +/- s.e.m.).

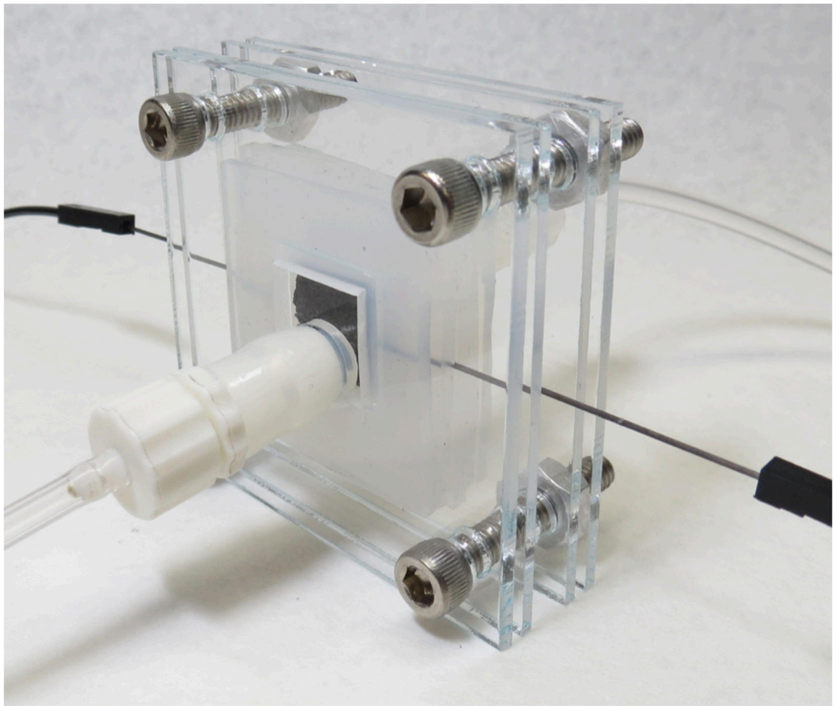

Supplementary Figure 10 - FT-CDI cell housing a single pair of bread electrodes. FT cell was fabricated using laser-cut acrylic and Ecoflex gaskets.

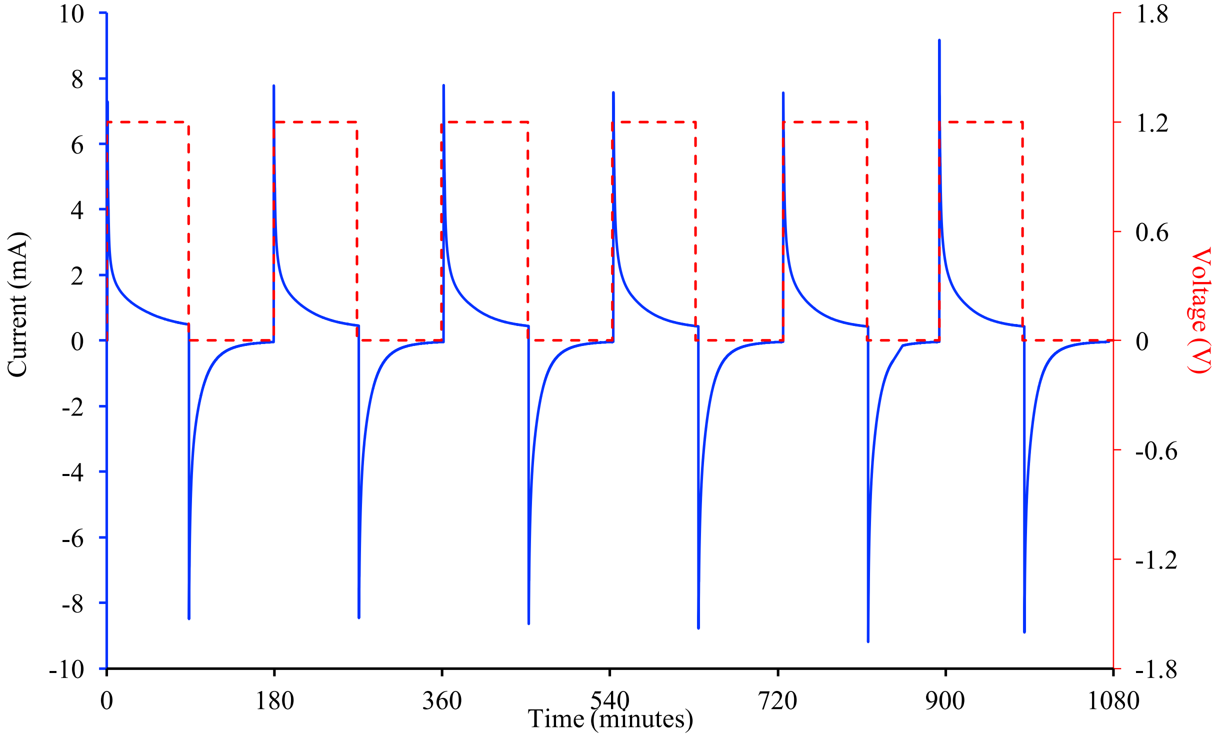

Supplementary Figure 11 – Current response and voltage profile for cyclic FT-CDI experiments with bread-derived electrodes. The profiles correspond to changes in salt concentration due to ion adsorption and ion desorption from Figure 4a.

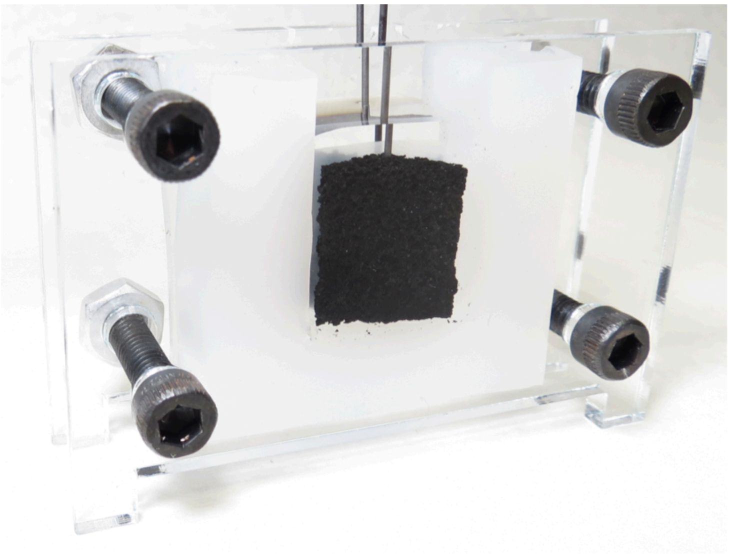

Supplementary Figure 12 - Housing device for solar-powered desalination with bread electrodes fabricated using fire. Thin nylon membrane in between the electrodes was used to prevent electrical shorting.
